# Supplementary material for: COZOID: contact zone identifier for visual analysis of protein-protein interactions
Source: BMC Bioinformatics. 2018 Apr 6;19:125. doi: 10.1186/s12859-018-2113-6 (PMC5889581; doi:10.1186/s12859-018-2113-6)
Supplement: Supplementary file 2 — Software build. Executable binary file of the software tool. (ZIP 118,784 kb) [file 12859_2018_2113_MOESM2_ESM.zip › cozoid/java/docs/beansbinding-1.2.1-doc.zip!/org/jdesktop/el/impl/ValueExpressionLiteral.html]

ValueExpressionLiteral (Beans Binding)


---


|  |  |  |
| --- | --- | --- |
| **Overview** \| **Package** \| **Class** \| **Use** \| **Tree** \| **Deprecated** \| **Index** \| **Help** | |  |
| **PREV CLASS**   NEXT CLASS | **FRAMES**    **NO FRAMES**     **All Classes** |  |
| SUMMARY: NESTED \| FIELD \| CONSTR \| METHOD | DETAIL: FIELD \| CONSTR \| METHOD |  |


---


## org.jdesktop.el.impl Class ValueExpressionLiteral

```
java.lang.Object
  org.jdesktop.el.Expression
      org.jdesktop.el.ValueExpression
          org.jdesktop.el.impl.ValueExpressionLiteral
```

**All Implemented Interfaces:**: java.io.Externalizable, java.io.Serializable

---

``` public final class ValueExpressionLiteral extends ValueExpression implements java.io.Externalizable ```

**See Also:**: Serialized Form

---

**Nested Class Summary**

**Nested classes/interfaces inherited from class org.jdesktop.el.Expression**

`Expression.ResolvedProperty, Expression.Result`


**Constructor Summary**

`ValueExpressionLiteral()`

`ValueExpressionLiteral(java.lang.Object value,
java.lang.Class expectedType)`


| **Method Summary** | |
| --- | --- |
| `boolean` | `equals(java.lang.Object obj)`             Determines whether the specified object is equal to this `Expression`. |
| `boolean` | `equals(ValueExpressionLiteral ve)` |
| `java.lang.Class` | `getExpectedType()`             Returns the type the result of the expression will be coerced to after evaluation. |
| `java.lang.String` | `getExpressionString()`             Returns the original String used to create this `Expression`, unmodified. |
| `java.lang.Class` | `getType(ELContext context)`             Evaluates the expression relative to the provided context, and returns the most general type that is acceptable for an object to be passed as the `value` parameter in a future call to the `ValueExpression.setValue(org.jdesktop.el.ELContext, java.lang.Object)` method. |
| `java.lang.Object` | `getValue(ELContext context)`             Evaluates the expression relative to the provided context, and returns the resulting value. |
| `int` | `hashCode()`             Returns the hash code for this `Expression`. |
| `boolean` | `isLiteralText()`             Returns whether this expression was created from only literal text. |
| `boolean` | `isReadOnly(ELContext context)`             Evaluates the expression relative to the provided context, and returns `true` if a call to `ValueExpression.setValue(org.jdesktop.el.ELContext, java.lang.Object)` will always fail. |
| `void` | `readExternal(java.io.ObjectInput in)` |
| `void` | `setValue(ELContext context, java.lang.Object value)`             Evaluates the expression relative to the provided context, and sets the result to the provided value. |
| `void` | `writeExternal(java.io.ObjectOutput out)` |

**Methods inherited from class org.jdesktop.el.ValueExpression**

`getResult, getSource, setSource`

**Methods inherited from class java.lang.Object**

`clone, finalize, getClass, notify, notifyAll, toString, wait, wait, wait`

**Constructor Detail**

### ValueExpressionLiteral

```
public ValueExpressionLiteral()
```

---


### ValueExpressionLiteral

```
public ValueExpressionLiteral(java.lang.Object value,
                              java.lang.Class expectedType)
```


**Method Detail**

### getValue

```
public java.lang.Object getValue(ELContext context)
```

:   **Description copied from class: `ValueExpression`**
:   Evaluates the expression relative to the provided context, and
    returns the resulting value.

    The resulting value is automatically coerced to the type
    returned by `getExpectedType()`, which was
    provided to the `ExpressionFactory` when this
    expression was created.

    :   **Specified by:**: `getValue` in class `ValueExpression`
    :   **Parameters:**: `context` - The context of this evaluation. **Returns:**: The result of the expression evaluation.

---


### setValue

```
public void setValue(ELContext context,
                     java.lang.Object value)
```

:   **Description copied from class: `ValueExpression`**
:   Evaluates the expression relative to the provided context, and
    sets the result to the provided value.

    :   **Specified by:**: `setValue` in class `ValueExpression`
    :   **Parameters:**: `context` - The context of this evaluation.: `value` - The new value to be set.

---


### isReadOnly

```
public boolean isReadOnly(ELContext context)
```

:   **Description copied from class: `ValueExpression`**
:   Evaluates the expression relative to the provided context, and
    returns `true` if a call to `ValueExpression.setValue(org.jdesktop.el.ELContext, java.lang.Object)` will
    always fail.

    :   **Specified by:**: `isReadOnly` in class `ValueExpression`
    :   **Parameters:**: `context` - The context of this evaluation. **Returns:**: `true` if the expression is read-only or `false` if not.

---


### getType

```
public java.lang.Class getType(ELContext context)
```

:   **Description copied from class: `ValueExpression`**
:   Evaluates the expression relative to the provided context, and
    returns the most general type that is acceptable for an object to be
    passed as the `value` parameter in a future call
    to the `ValueExpression.setValue(org.jdesktop.el.ELContext, java.lang.Object)` method.

    This is not always the same as `getValue().getClass()`.
    For example, in the case of an expression that references an
    array element, the `getType` method will return the
    element type of the array, which might be a superclass of the type
    of the actual element that is currently in the specified
    array element.

    :   **Specified by:**: `getType` in class `ValueExpression`
    :   **Parameters:**: `context` - The context of this evaluation. **Returns:**: the most general acceptable type; otherwise undefined.

---


### getExpectedType

```
public java.lang.Class getExpectedType()
```

:   **Description copied from class: `ValueExpression`**
:   Returns the type the result of the expression will be coerced to
    after evaluation.

    :   **Specified by:**: `getExpectedType` in class `ValueExpression`
    :   **Returns:**: the `expectedType` passed to the `ExpressionFactory.createValueExpression` method that created this `ValueExpression`.

---


### getExpressionString

```
public java.lang.String getExpressionString()
```

:   **Description copied from class: `Expression`**
:   Returns the original String used to create this `Expression`,
    unmodified.

    This is used for debugging purposes but also for the purposes
    of comparison (e.g. to ensure the expression in a configuration
    file has not changed).

    This method does not provide sufficient information to
    re-create an expression. Two different expressions can have exactly
    the same expression string but different function mappings.
    Serialization should be used to save and restore the state of an
    `Expression`.

    :   **Specified by:**: `getExpressionString` in class `Expression`
    :   **Returns:**: The original expression String.

---


### equals

```
public boolean equals(java.lang.Object obj)
```

:   **Description copied from class: `Expression`**
:   Determines whether the specified object is equal to this
    `Expression`.

    The result is `true` if and only if the argument is
    not `null`, is an `Expression` object that
    is the of the same type (`ValueExpression` or
    `MethodExpression`), and has an identical parsed
    representation.

    Note that two expressions can be equal if their expression
    Strings are different. For example, `${fn1:foo()}`
    and `${fn2:foo()}` are equal if their corresponding
    `FunctionMapper`s mapped `fn1:foo` and
    `fn2:foo` to the same method.

    :   **Specified by:**: `equals` in class `Expression`
    :   **Parameters:**: `obj` - the `Object` to test for equality. **Returns:**: `true` if `obj` equals this `Expression`; `false` otherwise. **See Also:**: `Hashtable`, `Object.equals(java.lang.Object)`

---


### equals

```
public boolean equals(ValueExpressionLiteral ve)
```

---


### hashCode

```
public int hashCode()
```

:   **Description copied from class: `Expression`**
:   Returns the hash code for this `Expression`.

    See the note in the `Expression.equals(java.lang.Object)` method on how two expressions
    can be equal if their expression Strings are different. Recall that
    if two objects are equal according to the `equals(Object)`
    method, then calling the `hashCode` method on each of the
    two objects must produce the same integer result. Implementations must
    take special note and implement `hashCode` correctly.

    :   **Specified by:**: `hashCode` in class `Expression`
    :   **Returns:**: The hash code for this `Expression`. **See Also:**: `Expression.equals(java.lang.Object)`, `Hashtable`, `Object.hashCode()`

---


### isLiteralText

```
public boolean isLiteralText()
```

:   **Description copied from class: `Expression`**
:   Returns whether this expression was created from only literal text.

    This method must return `true` if and only if the
    expression string this expression was created from contained no
    unescaped EL delimeters (`${...}` or
    `#{...}`).

    :   **Specified by:**: `isLiteralText` in class `Expression`
    :   **Returns:**: `true` if this expression was created from only literal text; `false` otherwise.

---


### writeExternal

```
public void writeExternal(java.io.ObjectOutput out)
                   throws java.io.IOException
```

:   **Specified by:**: `writeExternal` in interface `java.io.Externalizable`
:   **Throws:**: `java.io.IOException`

---


### readExternal

```
public void readExternal(java.io.ObjectInput in)
                  throws java.io.IOException,
                         java.lang.ClassNotFoundException
```

:   **Specified by:**: `readExternal` in interface `java.io.Externalizable`
:   **Throws:**: `java.io.IOException`: `java.lang.ClassNotFoundException`


---


|  |  |  |
| --- | --- | --- |
| **Overview** \| **Package** \| **Class** \| **Use** \| **Tree** \| **Deprecated** \| **Index** \| **Help** | |  |
| **PREV CLASS**   NEXT CLASS | **FRAMES**    **NO FRAMES**     **All Classes** |  |
| SUMMARY: NESTED \| FIELD \| CONSTR \| METHOD | DETAIL: FIELD \| CONSTR \| METHOD |  |


---
